# Queen Bee Larva, an Edible By-Product of Royal Jelly, Alleviate D-Galactose-Induced Aging in Mouse by Regulating Gut Microbiota Structure and Amino Acid Metabolism

**DOI:** 10.3390/antiox13111275

**Published:** 2024-10-22

**Authors:** Tong Zhao, Xiaofeng Xue, Pingxiang Liu, Han Hu, Kai Wang, Yutao Wang, Liming Wu

**Affiliations:** 1 Shandong Provincial Key Laboratory of Test Technology on Food Quality and Safety, Institute of Quality Standard and Testing Technology for Agro-Products, Shandong Academy of Agricultural Sciences, Jinan 250100, China; m13583224087_1@163.com (T.Z.); liupingxiangtri@163.com (P.L.); 2 Institute of Apicultural Research, Chinese Academy of Agricultural Sciences (CAAS), Beijing 100093, China; xue_xiaofeng@126.com (X.X.); huhan@caas.cn (H.H.); kaiwang628@gmail.com (K.W.); 3 Cooperative of Vegetable and Grain Cultivation, Liaocheng Yifeng Bloc, Liaocheng 252000, China

**Keywords:** queen bee larva, anti-aging, gut microbiota, metabolomics, amino acids

## Abstract

Queen bee larva (QBL), as a by-product of royal jelly, is a kind of protein-rich edible insect. However, the development and utilization of QBL have been very limited for an extended period, resulting in considerable economic waste. Notably, QBL has substantial potential for anti-aging treatments; however, systematic studies have been scarce. The present study aimed to analyze the effects of freeze-dried QBL powder (QBLP) treatment in a D-galactose (D-gal)-induced-aging mouse and to explore the mechanisms. A behavioral test indicated that QBLP-treated mice had improved cognitive function and memory decline caused by aging compared to untreated aged mice. Furthermore, QBLP treatment improved organ index in aged mice and prevented pathological damage to the brain tissue. Concomitantly, treatment of D-gal-induced-aging mice with QBLP significantly reduced the oxidative damage of serum and increased the skin moisture content of aging mice. Finally, integrated analyses of the gut microbiota and the serum metabolome showed that QBLP supplementation altered the composition of the gut microbiota, enriched biochemical pathways associated with amino acid metabolism, and adjusted serum concentrations of beneficial free amino acids. Overall, QBLP can improve symptoms related to D-gal-induced aging in mice by regulating gut microbiota structure and amino acid metabolism.

## 1. Introduction

Aging is a process comprising irreversible degenerative changes in various organs [1]. This process can lead to a range of disorders, including lung disease, diabetes, and malignancies [2,3]. In 2019, ~9% of the world’s population was classified as elderly (>65 years of age); this proportion is continuously increasing and is expected to reach 16% by 2050 [4]. As a result, there is also a continual increase in the demand for anti-aging healthcare products. Substantial efforts and monetary investments have therefore been dedicated to the research of pharmaceutical interventions for aging and age-related ailments. However, these efforts have yielded few achievements, and the potential side effects of drugs for age-related conditions have prevented their widespread adoption [5].

In comparison to pharmaceutical therapies, diet-related interventions tend to be safer and to focus on disease prevention rather than treatment. Over the past decade, there has been a notable shift in the focus of research into aging and age-related ailments toward utilization of natural bioactive compounds [6]. Such strategies can enhance an individual’s health by optimizing energy metabolism, nutrient access, and microbial community function [7]. For example, edible roses, which have been used in traditional medicine for over a thousand years to treat various diseases, are highly biologically active and have anti-aging, anti-hypertension, hepatoprotective, and other beneficial effects [8,9]. Probiotic supplements may exert anti-aging effects through modulation of the gut microbiota [10]. Studies in this area are important for their contributions to prolonging the healthy human lifespan.

Queen bee larva (QBL) is harvested as a by-product of royal jelly production. In the process of royal jelly production, QBL are removed and discarded [11]. In fact, QBL are edible insects with extremely high nutritional value, with their dry matter containing up to 50% protein [12,13,14]. Furthermore, QBL have beneficial effects on human health, including the alleviation of insomnia and related neurotransmitter disorders [13]. However, the ameliorative effects of QBL on aging in vivo and the underlying mechanisms remain unclear.

Imbalances or disruptions in the gut microbiota are considered significant indicators of human aging. For example, decreases in the relative abundance of Bacteroidota are characteristic of aging individuals [15]. Metabolomics methods are commonly used to assess the metabolic effects of dietary interventions. An integrated approach that encompasses both gut microbiota and metabolome assessment is therefore a robust strategy for investigating the impacts of diet-based therapies [16]. In the present study, we used a mouse model of aging to assess the putative anti-aging activities of QBL powder (QBLP). The specific pathways associated with the anti-aging effects of QBLP were assessed with a combined gut microbial sequencing and metabolomic analysis. This study was designed to clearly demonstrate the anti-aging effects of QBLP in multiple dimensions, promoting future applications of QBLP in anti-aging therapies to improve quality of life during the aging process.

## 2. Materials and Methods

### 2.1. Materials and Reagents

QBLP was obtained from Yunnan Laodianhuang Food Co., Ltd. (Kunming, China). The production method was described in a previous study [11,13]. Met (>98% purity), D-gal (>98% purity), carboxymethyl cellulose sodium (CMC-Na), and amino acid standards were purchased from Yuanye Biotechnology Co. Ltd. (Shanghai, China). Ortho-phthalaldehyde (OPA) and 9-fluorenylmethyl chloroformate (FMOC) were purchased from Agilent Technologies (Santa Clara, CA, USA).

### 2.2. Animal Care and Treatment

The experimental protocol was reviewed and approved by the Animal Ethics Committee of the Institute of Apicultural Research of the Chinese Academy of Agricultural Sciences (Beijing, China) (AEC NO: 20210531). The animal experiments were performed in the Barrier Laboratory of Zhejiang Chinese Medical University Laboratory Animal Research Center (Hangzhou, Zhejiang, China, ACE code: IACUC-20211025-16). All mouse experiments were conducted following the ARRIVE guidelines for ethical use and care of animals in research (Animal Certificate No. SCXK (HU) 2017-0012).

Thirty female C57BL/6J mice (18–22 g each) were raised in the laboratory. The mice were randomly assigned to each of five groups (six mice per group): blank control (G0), aging model (G1), Met treatment (G2), QBLP-L treatment (G3), and QBLP-H treatment (G4) [17]. Mice in groups G1–G4 received 100 mg/kg/d D-gal via injections in the neck each day for eight weeks; the G0 group was injected with 0.9% saline for the same duration [18]. At the same time as saline or D-gal was injected, groups G0–G4 were orally administered 5% CMC-Na, 5% CMC-Na, 100 mg/kg/d Met, low-dose QBLP (1 g/kg/d), or high-dose QBLP (5 g/kg/d), respectively. Further experiments began after the 8-week treatment period [19].

### 2.3. Cognitive Analysis

Morris water maze tests were performed as previously described [20,21]. The mice were trained four times each day for the first four days. On the fifth day of the experiment, space exploration trials were performed. First, the hidden platform was placed in the pool, and the mice were situated in the first quadrant pool for 60 s. The number of platform crossings and the latency of spatial behavior were recorded for each mouse for the duration of the swimming period.

### 2.4. Organ Index Analysis

At the conclusion of the experiment, all mice were fasted for 12 h then sacrificed via the cervical dislocation method. The brain, heart, kidney, liver, and spleen were collected and immediately weighed. The calculation method of organ index refers to Liao et al. [18].

### 2.5. Histopathology of the Brain

Brain tissues were transferred to Wuhan Servicebio Technology CO. LTD (Wuhan, China) for tissue sectioning and staining. The stained tissue slices were viewed under a DFC7000 T light microscope (Leica, Wetzlar, Germany) at 400× magnification to visually assess differences in cell appearance between treatment groups.

### 2.6. Evaluation of Serum Lipid Peroxidation

Fresh blood samples were collected from the sacrificed mice and stored at −80 °C prior to further analysis. Serum was collected via centrifugation at 1150× *g* (Centrifuge 5424R, Eppendorf, Hamburg, Germany) at 4 °C for 15 min. The levels of malondialdehyde (MDA) in the serum were measured with an MDA assay kit (Nanjing Jiancheng Bioengineering Institute, Nanjing, China) according to the manufacturer’s instruction.

### 2.7. Skin Moisture Measurement

Skin moisture was determined by GB/T5009.3-2010, China [22].

### 2.8. 16S rRNA Sequencing of the Gut Microbiota

Fresh feces were collected from the sacrificed mice and stored at −80 °C prior to further analyses [16]. The E.Z.N.A.^®^ Soil DNA kit (Omega Bio-tek, Norcross, GA, USA) was used to extract bacterial genomic DNA from the fecal samples. PCR was then conducted with barcoded conventional 338F (5′-ACTCCTACGGGAGGCAGCAG-3′) and 806R (5′-GGACTACHVGGGTWTCTAAT-3′) primers to amplify the V3–V4 hypervariable region of the bacterial 16S rRNA gene. Purified amplicons were pooled in equal quantities for paired-end sequencing on the Illumina MiSeq platform (San Diego, CA, USA) by Shanghai Majorbio Biotechnology Co. Ltd. (Shanghai, China) following their standard protocol.

The raw sequencing data were filtered for quality and the paired-end reads were merged. OTUs were called at the standard 97% threshold in Uparse v11.0 (http://www.drive5.com/uparse/, accessed on 27 December 2022). *α*-diversity indices, namely, the Chao, coverage, Shannon, and Simpson indices, were calculated in mothur (v1.30.2). *β*-diversity was assessed via PLS-DA in QIIME v1.9.1 [23].

After identifying differences in the microbial community structure between treatment groups, PICRUST analysis was performed to predict the functions associated with each microbial community [24].

### 2.9. Serum Metabolomic Analysis

#### 2.9.1. Sample Preparation

A total of 100 µL per serum sample was mixed with 400 µL of 1:1 methanol–acetonitrile (*v*/*v*) and oscillated for 30 s. The supernatant was removed, and 350 µL of 1:1 methanol–acetonitrile (*v*/*v*) was added to the sediment in the lower layer. Samples were oscillated for 2 min, sonicated at 30 kHz for 30 s in an ice water bath, then centrifuged at 18,407× *g* at 4 °C for 15 min. The supernatants from the two steps were combined for each sample then concentrated and dried under a vacuum. Each dried sample was dissolved in 200 μL of 1:1 methanol–acetonitrile (*v*/*v*) and sonicated at 30 kHz for 30 s in an ice water bath. Finally, samples were centrifuged at 13,523× *g* for 15 min, and the supernatants were collected for analysis.

#### 2.9.2. Untargeted Metabolomic Analysis

The ultra performance liquid chromatography (UPLC) system was connected to a Q-Exactive Orbitrap Mass Spectrometer (Thermo Fisher Scientific, Waltham, MA, USA) equipped with a heated electrospray ionization (HESI) probe. Separations were performed on a ZORBAX RRHD Eclipse Plus C18 column (150 mm × 3.0 mm, 1.8 μm) at 50 °C in positive and negative ionization modes. Gradient elution was performed using 0.1% formic acid acetonitrile solution (A) + 0.1% formic acid aqueous solution (B) at a flow rate of 0.3 mL/min and an injection volume of 4 μL. The gradient elution procedure was as follows: 0–2 min; 1% solution A 2–5 min; 1–25% A 5–20 min; 25–95% A 20–25 min; 95% A 25–26 min; 95–1% A 26–32 min, 1% A. The spray voltages were 3.5 kV and 3 kV in positive and negative modes, respectively. For mass spectrometry (MS), the sheath gas rate was 30 L/min; the auxiliary gas rate was 5 L/min; the capillary temperature was 320 °C; the mass range was 70–1000 *m/z*; and the mass resolution was 70,000. Spectrum analyses and compound identification were conducted in Compound Discoverer 3.3 (Thermo Fisher Scientific, Waltham, MA, USA).

#### 2.9.3. Amino Acid Analysis

Samples were derivatized with OPA and FMOC reagents then analyzed on the Agilent 1260 Infinity II Prime LC instrument using an Agilent AdvanceBio AAA C18 column (4.6 mm × 100 mm, 2.7 μm). The protocol was consistent with that described by Machado et al., except where otherwise specified below [25]. Fluorescence detection was monitored at λexc = 340 nm/λem = 450 nm (from 0.0 to 11.5 min) for OPA-derivatives and at λexc = 230 nm/λem = 305 nm (from 11.5 to 28 min) for FMOC-derivatives. The gradient elution program for each run was as follows: 2% B from 0 to 0.5 min; 57% B at 23.2 min; 57–100% B from 23.2 to 23.3 min; 100% B from 23.3 to 27.2 min; 2% B at 27.3 min; 2% B from 27.3 to 28 min [26].

### 2.10. Statistical Analysis

There were six biological replicates for all measurements. The mean and standard deviation were calculated within each treatment group for each measurement type. Differences between treatment groups were assessed via one-way analysis of variance (ANOVA) or a non-parametric test and were considered statistically significant at *p* < 0.05. Multivariate statistical analysis was conducted with the metabolite profiles using OPLS-DA. The OPLS-DA models were assessed with CV-ANOVA. Thresholds of VIP > 1 and *p* < 0.05 were used to classify metabolites as differentially abundant between treatment groups. The enrichment of specific biochemical pathways associated with differentially abundant metabolites was determined using data from the KEGG database (http://www.genome.jp/kegg, accessed on 20 November 2022).

## 3. Results

### 3.1. Effects of QBLP on the Learning Behaviors in Aging Mice

**Figure 1:**
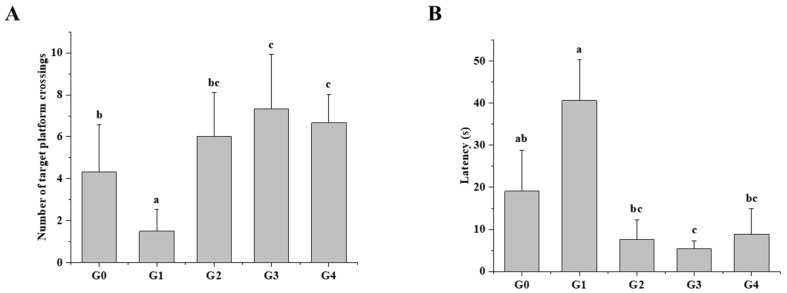
Morris water maze test results in a mouse model of aging. (**A**) Average platform-crossing frequency of mice in each group. G0, blank control; G1, aging mouse model induced with D-galactose; G2, induced-aging group treated with metformin hydrochloride; G3, induced-aging group treated with a low dose (1 g/kg/d) of queen bee larva powder (QBLP); G4, induced-aging group treated with a high dose (5 g/kg/d) of QBLP. (**B**) Average escape latency of mice in each group. N = 6. Different letters indicate significant differences between groups (*p* < 0.05).

To quantify the effects of D-galactose (D-gal)-induced aging in mice and potential improvements due to QBLP treatment, we first conducted a Morris water maze test (Figure 1). Induced-aging mice (G1) showed a significantly decreased frequency of platform crossing (*p* < 0.05) and a longer escape latency compared to blank control mice (G0), suggesting that they exhibited spatial memory impairment caused by D-gal-induced aging. Treatment of induced-aging mice with the control therapy (G2) led to significant improvements in both parameters (*p* < 0.05). Notably, there were no significant differences in escape latency or the frequency of platform crossing between mice given metformin hydrochloride (Met) and either dose of QBLP (*p* > 0.05).

### 3.2. Effects of QBLP on the Organ Indicators in Aging Mice

Compared with the G0 group, the brain index, cardiac index, kidney index, liver index, and spleen index of the G1 group decreased significantly (*p* < 0.05) (Table 1). Compared with the G1 group, the brain index, cardiac index, kidney index, liver index, and spleen index of QBLP treatment group (G3 and G4) significantly increased. However, high-dose supplementation of QBLP (G4) did not have a better therapeutic effect on the brain index, cardiac index, and kidney index of D-gal-induced-aging mice compared to low-dose supplementation of QBLP (G3) (*p* > 0.05).

### 3.3. Effects of QBLP on the Brain Tissues in Aging Mice

**Figure 2:**
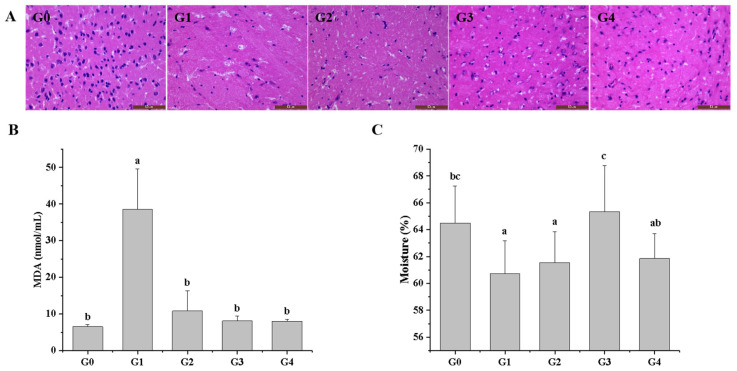
Protective effect of queen bee larva powder (QBLP) on D-galactose (D-gal)-induced-aging mice. (**A**) Brain slices stained with hematoxylin and eosin (HE) and viewed at 400× magnification. G0, blank control; G1, aging mouse model induced with D-gal; G2, induced-aging group treated with metformin; G3, induced-aging group treated with a low dose (1 g/kg/d) of QBLP; G4, induced-aging group treated with a high dose (5 g/kg/d) of QBLP. N = 6. Blue spots indicate nuclei. Scale bars = 50 μm. (**B**) Effects of QBLP on the contents of malondialdehyde in serum. (**C**) Effect of QBLP on skin moisture content. Different letters indicate significant differences between groups (*p* < 0.05).

We next analyzed the effects of aging and QBLP treatment on the brain in terms of cellular structure. Histological observations revealed plump, round nuclei in G0 samples, in contrast to the fuzzy nuclei in G1 samples (Figure 2A). Compared with the G1 group, the G3 and G4 samples contained fewer shrunken cells, more intact nuclei, and a greater volume of cytoplasm.

### 3.4. Effects of QBLP on the Antioxidant Property in Aging Mice

We determined the levels of malondialdehyde (MDA) in serum to evaluate the effect of QBLP on oxidative stress in aging mice. The results demonstrated that compared with the G0 group, the MDA level was abnormally increased in the G1 group, indicating that D-gal treatment reduced the oxidative defense of aging mice. D-gal-induced increases in serum MDA levels were ameliorated by QBLP or Met (*p* < 0.05) (Figure 2B).

### 3.5. Effects of QBLP on the Skin Moisture Content in Aging Mice

The G1 group showed a significant decrease in skin moisture compared to the G0 group (*p* < 0.05) (Figure 2C), indicating that D-gal accelerated the aging of mice skin. After 1 g/kg/d QBLP treatment, skin water content was significantly increased (*p* < 0.05). However, there were no significant differences between the G4 group and the G1 group (*p* > 0.05).

A series of results indicated that compared to low-dose supplementation of QBLP, high-dose supplementation of QBLP did not have a better improvement effect on aging mice, indicating that higher concentration of QBLP may have a side effect on mouse. Similar results have also been reported. For example, compared to 100 μM, 200 μM nomilin showed the lower lifespan extending effects on *Caenorhabditis elegans* [27]. Further experiments were therefore conducted with only G3 representing the QBLP treatment for simplicity.

### 3.6. Effects of QBLP on the Overall Structure and Composition of the Gut Microbiota in Aging Mice

**Figure 3:**
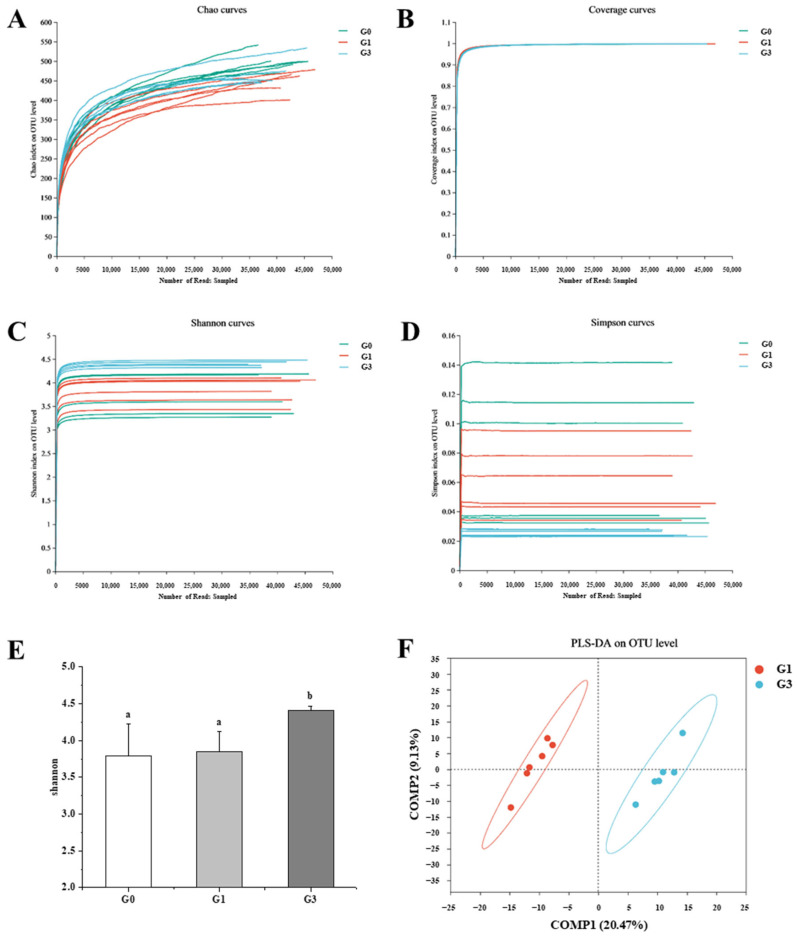
Impacts of queen bee larva powder (QBLP) treatment on the gut microbiota in a mouse model of aging. (**A**–**D**) α-diversity curves for the (**A**) Chao, (**B**) coverage, (**C**) Shannon, and (**D**) Simpson indices. (**E**) Shannon index values for each group. G0, blank control; G1, aging mouse model induced with D-galactose; G3, induced-aging mouse model treated with QBLP. Different letters indicate significant differences between groups (*p* < 0.05). (**F**) Partial least squares discriminant analysis (PLS-DA) score plot demonstrating between-sample (β) microbial diversity.

We next compared the gut microbiota of blank control mice (G0), induced-aging mice (G1), and induced-aging mice treated with QBLP (G3). Eighteen fecal samples (six mice in each of the three treatment groups) yielded a total of 947,254 high-quality reads. The rarefaction curves demonstrated convergence for all 18 samples (Figure 3A–D), indicating that the sequencing depth used here was sufficient to capture the microbial diversity accurately, including rare community members.

The microbial community structure was evaluated in terms of both *α* (within-sample) and *β* (between-sample) diversity. Shannon index values (which measure *α*-diversity) indicated that QBLP treatment significantly increased the gut microbiome diversity compared to the blank control and the untreated induced-aging mouse (Figure 3E, *p* < 0.05). Partial least squares discriminant analysis (PLS-DA) was used to visualize *β*-diversity G0 samples clustered away from the G1 samples (Figure S1). Furthermore, G3 samples showed a clear separation from G1 samples (Figure 3F, *p* < 0.05).

**Figure 4:**
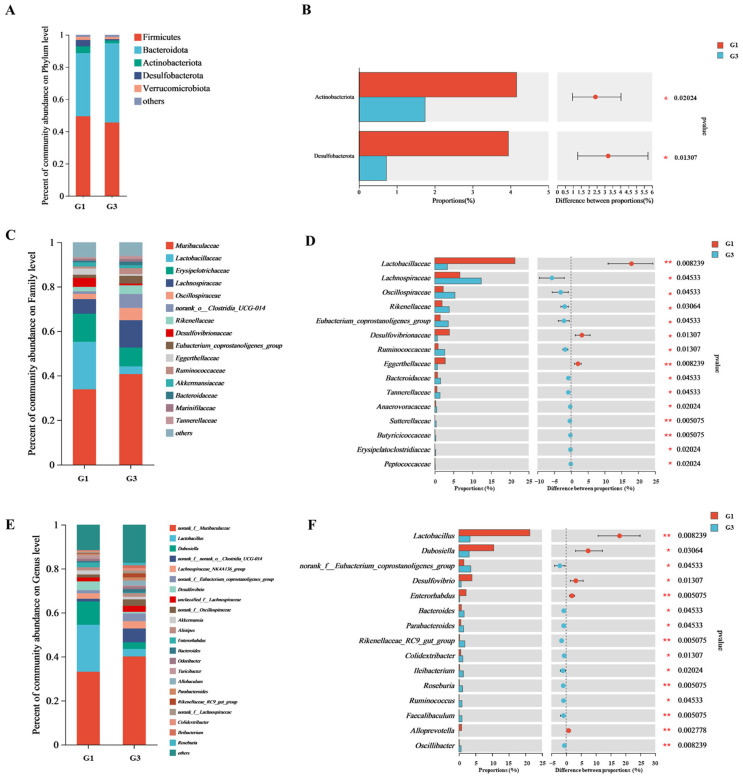
Effects of queen bee larva powder (QBLP) treatment on the intestinal microbial community in a mouse model of aging. (**A**,**C**,**E**) Relative abundances of the most dominant microbial (**A**) phyla, (**C**) families, and (**E**) genera in the gut microbiota of induced-aging mice (G1) and induced-aging mice treated with QBLP (G3). (**B**,**D**,**F**) Differences in the relative abundance of specific dominant microbial (**B**) phyla, (**D**) families, and (**F**) genera between the gut microbiota of G1 and G3 mice.

To further explore specific changes in the intestinal microflora composition due to QBLP treatment, we analyzed the gut microbiota of G1 and G3 samples at the phylum, family, and genus levels. At the phylum level, the microbial communities of both groups were dominated by Firmicutes and Bacteroidota, followed by Actinobacterota and Desulfobacterota (Figure 4A,B). Actinobacterota and Desulfobacterota were significantly less abundant in G3 than in G1 mice (*p* < 0.05). At the family level, QBLP-treated mice showed a significant increase in *Lachnospiraceae*, *Oscillospiraceae*, *Rikenellaceae*, and *Eubacterium_coprostanoligenes_group*, and a significant decrease in *Lactobacillaceae* and *Desulfovibrionaceae* compared with induced-aging mice (Figure 4C,D, *p* < 0.05 or 0.01). At the genus level, QBLP treatment significantly increased the abundance of *norank_f_Eubacterium_coprostanoligenes_group*, *Bacteroides*, *Parabacteroides*, *Rikenellaceae_RC9_gut_group*, *Colidextribacter*, *Ileibacterium*, *Roseburia*, *Ruminococcus*, *Faecalibaculum*, and *Oscillibacter* (*p* < 0.05 or 0.01). Notably, the relative abundances of *Lactobacillus*, *Dubosiella*, *Desulfovibrio*, *Enterorhabdus*, and *Alloprevotella* were all significantly decreased in response to QBLP treatment (Figure 4E,F, *p* < 0.05 or 0.01).

With the entire microbial community of each sample included, there were 302 metabolic pathways present in the gut microbiota. Using a threshold of 0.1% abundance to retain only operational taxonomic units (OTUs) with significant contributions to the microbial community, there were 114 metabolic pathways present, including 46 that were significantly enriched or depleted in G1 compared to G3 samples (Figure S2). Of these 46 differentially present metabolic pathways, 10 were involved in amino acid metabolism.

### 3.7. Effects of QBLP on Serum Metabolite Profiles in Aging Mice

**Figure 5:**
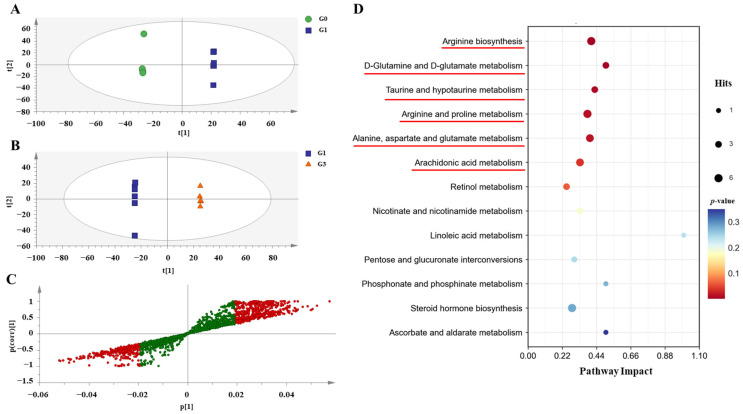
Treatment with queen bee larva powder (QBLP) altered serum metabolite profiles in a mouse model of aging. (**A**,**B**) Orthogonal partial least squares discriminant analysis (OPLS-DA) score plots representing serum metabolite profiles. This analysis showed distinct clustering of untreated induced-aging mice (G1) away from (**A**) blank control mice (G0) and (**B**) induced-aging mice treated with QBLP (G3). (**C**) S-plot of OPLS-DA model between group G1 and G3. Red dots indicate variable importance in prediction (VIP) > 1 and *p* < 0.05. (**D**) Analysis of potential biomarker pathways in aging mice induced with D-galactose. Underline indicates metabolic pathways with pathway effects > 0.2 and *p* < 0.05.

To gain a more comprehensive understanding of the mechanism by which QBLP ameliorated the effects of aging, a non-targeted metabolomics approach was applied to serum samples from G0, G1, and G3 samples. The verall differences between samples were assessed with orthogonal PLS-DA (OPLS-DA). The results of cross-validation-analysis of variance (CV-ANOVA) determined that the OPLS-DA model was statistically significant (Table S1) [28]. G1 samples predominantly clustered together away from both G0 (Figure 5A) and G3 (Figure 5B) samples. This indicated substantial alterations in serum metabolite profiles due to an aging-induced disordered metabolism.

We next screened metabolites that were differentially abundant between the serum samples of mice in the G1 and G3 groups based on variable importance in projection (VIP) scores [16]. Using thresholds of VIP > 1 and *p* < 0.05, there were 784 differentially abundant metabolites between G1 and G3 samples (Figure 5C). To identify the metabolic pathways in which those metabolites were involved, the enriched metabolites were examined with Kyoto Encyclopedia of Genes and Genomes (KEGG) biochemical pathway analysis. In total, metabolites that were differentially abundant between G1 and G3 samples were identified as members of 46 metabolic pathways. Using a more stringent threshold (pathway impact > 0.2 and *p* < 0.05) yielded six enriched metabolic pathways that may have been associated with minimization of aging-related symptoms through QBLP treatment: arginine biosynthesis; D-glutamine and D-glutamate metabolism; taurine and hypotaurine metabolism; arginine and proline metabolism; alanine, aspartate, and glutamate metabolism; and arachidonic acid metabolism (Figure 5D). Notably, five of the six significantly enriched pathways were associated with amino acid metabolism. Taurine is a sulfur-containing amino acid that can be derived from the metabolism of methionine and cysteine; studies have reported that taurine and hypotaurine possess antioxidant properties, which are closely related to anti-aging [29]. These findings were consistent with the predicted differences in gut microbial functions between treatment groups.

### 3.8. Effects of QBLP on Free Amino Acid Concentrations in the Serum

**Figure 6:**
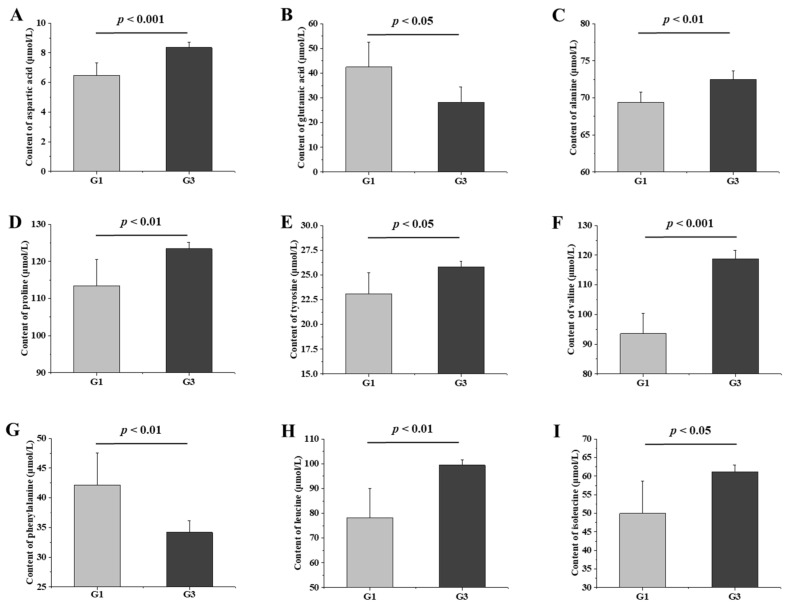
Effects of queen bee larva powder (QBLP) treatment on serum free amino acid contents in a mouse model of aging: (**A**–**I**) Levels of (**A**) aspartic acid; (**B**) glutamic acid; (**C**) alanine; (**D**) proline; (**E**) tyrosine; (**F**) valine; (**G**) phenylalanine; (**H**) leucine; and (**I**) isoleucine. G1, aging mouse model induced with D-galactose; G3, induced-aging mice treated with QBLP.

The initial results of this study suggest that QBLP-mediated amelioration of aging-related effects was associated with amino acid metabolism. To establish this more directly, we measured the free amino acid concentrations in serum samples from G1 and G3 groups. Consistent with our prior findings, the analysis revealed significant differences in concentrations of nine amino acids between the two groups. Specifically, QBLP treatment significantly increased contents of aspartic acid, alanine, proline, tyrosine, valine, leucine, and isoleucine and significantly decreased levels of glutamic acid and phenylalanine (Figure 6, *p* < 0.05 or 0.01 or 0.001).

**Figure 7:**
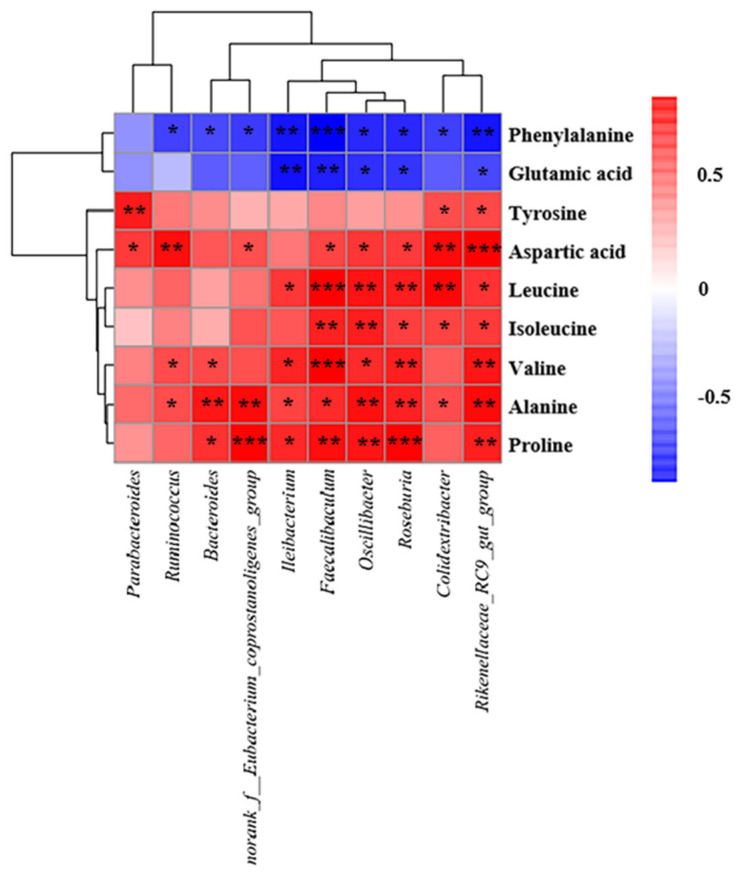
Correlations between serum free amino acid levels and gut microbial taxon abundance in a mouse model of aging. (∗) *p* < 0.05, (∗∗) *p* < 0.01, and (∗∗∗) *p* < 0.001 according to Spearman’s rank correlation coefficient.

We next sought to identify potential relationships between treatment-related differences in the gut microbiota and in amino acid metabolism. Spearman correlation analysis showed that of the 10 bacterial genera that were significantly more abundant after QBLP treatment, nine were negatively correlated with phenylalanine levels and five were negatively correlated with glutamic acid at a threshold of *p* < 0.05 (Figure 7) [30]. There were many more positive correlations: nine genera with alanine; eight with aspartic acid; seven each with proline and valine; six with leucine; and five with isoleucine. These results confirmed that QBLP-mediated alterations in the gut microbiota corresponded to significant changes in the serum metabolite profiles.

## 4. Discussion

Due to a widespread increase in human longevity, anti-aging has been an increasing focus of both basic and applied research [5]. There is a complex relationship between reactive oxygen species (ROS), antioxidants, and aging. Accumulation of ROS and an imbalance in oxidative defense can lead to organismal aging [31]. Indeed, a well-characterized model of aging in mice was generated through D-gal injections, these reportedly caused excessive ROS production, inducing aging-related effects such as cellular damage, mitochondrial dysfunction, and inflammation of the brain [18].

Edible insects have attracted a great deal of research attention as potential anti-aging treatments. For example, products containing *Clanis bilineata* larvae can delay aging by suppressing oxidative damage and are used as a dietary supplement in the food industry [32]. Compounds derived from *Tenebrio molitor* larvae also have the potential to delay aging-related disorders such as Alzheimer’s disease [33]. Although QBL is a by-product, it is also a high-quality edible insect that is rich in protein [13]. Previous studies have shown that QBLP can delay aging in *Caenorhabditis elegans* [11], but its effects on mammalian tissues and organs have remained unclear, as have the related anti-aging mechanisms at the molecular level.

To better understand the possible anti-aging effects of QBLP, we here analyzed a suite of parameters in the D-gal-induced mouse aging model: cognitive dysfunction and memory; organ indicators; brain health at the morphological and cellular levels; antioxidant property of serum; gut microbial community structure; and serum metabolite profiles. QBLP treatment was found to improve cognitive function and memory decline caused by aging (Figure 1), as well as to improve the brain index, cardiac index, kidney index, liver index, and spleen index (Table 1). Histopathological examination revealed an increase in necrotic cells within the brain tissues of untreated aging mice. However, QBLP treatment inhibited this effect (Figure 2A), demonstrating a protective effect on brain tissue. Zhang et al. similarly showed that treatment with an enzymatic polysaccharide derived from *Pleurotus eryngii* residue has anti-aging and antioxidant effects on mouse brain tissues [34]. Oxidative stress is closely related to the aging process. The increase in the MDA level indicated that D-Gal caused damage to the antioxidant defense system, while QBLP treatment meaningfully reduced the oxidative damage (Figure 2B). Skin moisture can reflect the process of skin aging and decreases gradually with aging. Skin moisture content showed improvement (*p* < 0.05) with 1 g/kg/d QBLP treatment (Figure 2C). Based on these findings, we hypothesize that QBLP could improve a variety of symptoms and performances during the aging process. However, additional studies characterizing these parameters in greater detail will be necessary to confirm these hypotheses.

The mammalian gut microbiota is composed of trillions of microorganisms. It is also known as the “second human genome” and is sometimes considered a type of functional organ in its own right [35]. The gut microbial community has a critical role in modulating the host immune system and energy metabolism [16]. Furthermore, the gut microbiota can influence host lifespan [36,37,38,39]. Numerous studies have indicated that the gut microbiota is susceptible to perturbation by a wide variety of factors, including diet, environment, and medications [40,41,42]. Dietary intake is regarded as the primary factor affecting the gut microbial community membership and structure [43]. For example, host diet explains up to 60% of variation in the gut microbiota, whereas genetic factors explain < 12% [44]. Furthermore, studies have revealed key associations between aging and the gut microbiome, such as age-related increases in Proteobacterial species abundance [45].

In the present study, we found that QBLP treatment effectively altered OTU abundance in the mouse intestinal microflora (Figure 3). Although the gut microbiota was dominated by four phyla (Actinobacterota, Bacteroidota, Desulfobacterota, and Firmicutes) in mice of all groups, mice treated with QBLP showed a higher relative abundance of Bacteroidota and a lower proportion of Firmicutes (Figure 4A). This was consistent with a prior publication showing that selenium-enriched *Chrysomyia megacephala* (Fabricius) larvae powder increased the abundance of Bacteroidota in D-galactose induced-aging mice and this prevented toxins from entering the blood by increasing intestinal wall thickness, reducing oxidative stress [46]. *Ruminococcaceae* and *Lachnospiraceae* are core bacterial families found in animal digestive tracts, and members of these families decrease in abundance with age [47]. Importantly, we here discovered that QBLP supplementation significantly increased the relative abundance of *Ruminococcaceae* and *Lachnospiraceae* in the guts of aging mice, demonstrating a reversal of the trends typically observed with aging (Figure 4D, *p* < 0.05). Furthermore, QBLP treatment significantly increased the abundance of the genera *Rikenellaceae*, *Eubacterium*, *Ruminococcus*, *Roseburia*, and *Oscillibacter* (Figure 4F, *p* < 0.05 or 0.01). Increased *Rikenellaceae* abundance can improve muscle mass and promote longevity in mice by reducing visceral fat accumulation [48,49]. Recent studies have identified *Eubacterium* and *Ruminococcus* as potentially beneficial members of the intestinal flora [50,51]. Prior studies have indicated that many species in the genus *Roseburia* can generate short chain fatty acids (SCFAs), and this genus is therefore considered beneficial to human health [52]. *Oscillibacter* is the primary SCFA-producing bacterial genus in the gut and increases in the abundance of *Oscillibacter* spp. can promote the health of the intestinal flora in mice [53]. These results together indicate that QBLP-mediated amelioration of the aging phenotype in mice was partially attributable to regulation of the structure and composition of the gut microbiota, specifically via enrichment of beneficial families and genera.

Imbalances in the intestinal flora are considered a primary factor leading to metabolic disorders [54]. We here conducted functional analyses of microbial communities to link microbial community structure with metabolic outcomes. QBLP treatment was found to enrich multiple metabolic pathways, among which ten were involved in amino acid (Figure S2). To assess the impacts of QBLP on aging mice more directly, serum metabolite profiles were analyzed with an untargeted approach, which revealed significant differences in the serum metabolomes. Consistent with the microbial community functional predictions, the greatest differences were in metabolites involved in amino acid metabolism (Figure 5D).

Here, QBLP treatment was found, respectively, to increase and decrease the serum levels of seven and two amino acids significantly (Figure 6). Numerous investigations have previously substantiated the close association between amino acid levels and the aging process [55]. Liuwei Dihuang decoction, a traditional Chinese medicine formula, has anti-aging effects in 20-month-old naturally aging mice, and Liuwei Dihuang decoction regulates proline metabolism, helping to alleviate the effects of aging [56]. Another classic traditional Chinese medicine formula, Guilingji, improves cognitive function and memory capabilities in naturally aging rats by regulating arginine and proline metabolism [57]. Similarly, we here found that QBLP supplementation reduced aging-related symptoms and altered proline metabolism in aging mice (Figure 6D). Reductions in tyrosine content can affect neurotransmitter production and ultimately cause a variety of age-related diseases, such as Parkinson’s disease [58]. Here, QBLP supplementation increased free tyrosine contents in the serum of D-gal-induced-aging mice (Figure 6E). Thus, QBLP could serve as a treatment for neurodegenerative diseases caused by tyrosine deficiency. In addition to serving as protein building blocks, amino acids are involved in a range of biochemical pathways and functions. For example, glutamate functions as a critical excitatory neurotransmitter in the cerebral cortex. At high concentrations, it can cause excitatory cell damage and mitochondrial dysfunction. Elevated glutamate levels are a primary cause of cell injury because of its potent capacity to increase intracellular calcium levels through ionotropic receptors [59]. We here found that QBLP supplementation decreased glutamate content in the serum of D-gal-induced-aging mice, indicating that QBLP has the potential to mitigate glutamate-induced excitatory cell damage (Figure 6B). Other amino acids are also known to directly affect health in animals. Valine, leucine, and isoleucine are branched-chain amino acids (BCAAs), which play important roles in maintaining the integrity of the intestinal barrier and in protecting the permeability of the intestinal mucosa [60]. BCAA concentrations were significantly higher in the sera of mice treated with QBLP than in the untreated induced-aging group, suggesting that QBLP treatment could reduce intestinal epithelial cell inflammation and pathological intestinal damage caused by D-gal-induced aging (Figure 6F,H,I) [61]. Importantly, significant alterations to the gut microbiota as a result of QBLP treatment were correlated with alterations abundance of key amino acids in the sera (Figure 7). Overall, these results indicated that QBLP could exert anti-aging functions by regulating amino acid metabolism and structure of gut microbiota in mice.

## 5. Conclusions

In total, these findings demonstrate that QBL, a by-product of royal jelly, has anti-aging effects via a mechanism related to adjusting the balance of gut microbiota and amino acid metabolism. Although further research is needed to clarify whether QBL has positive effects on normally aging mice and to identify the anti-aging components within QBL, this work lays a strong research foundation for the future application of QBL as a dietary supplement to promote a prolonged healthy human lifespan throughout the world.

## Figures and Tables

**Table 1:** Effects of queen bee larva powder (QBLP) on organ index in D-galactose-treated mice.

Group	Brain Index (mg/g)	Cardiac Index (mg/g)	Kidney Index (mg/g)	Liver Index (mg/g)	Spleen Index (mg/g)
G0	21.58 ± 0.62 ^b^	4.85 ± 0.22 ^c^	5.38 ± 0.41 ^bc^	36.29 ± 1.32 ^c^	2.84 ± 0.23 ^bc^
G1	19.15 ± 1.30 ^a^	4.22 ± 0.36 ^a^	4.93 ± 0.28 ^a^	32.62 ± 1.35 ^a^	2.18 ± 0.16 ^a^
G2	21.71 ± 0.86 ^b^	4.33 ± 0.13 ^ab^	5.18 ± 0.25 ^ab^	37.75 ± 1.61 ^c^	2.57 ± 0.15 ^ab^
G3	22.05 ± 0.66 ^bc^	4.75 ± 0.42 ^c^	5.37 ± 0.12 ^bc^	37.49 ± 0.76 ^c^	2.72 ± 0.21 ^b^
G4	22.88 ± 0.62 ^c^	4.66 ± 0.25 ^bc^	5.57 ± 0.25 ^c^	34.68 ± 1.19 ^b^	3.10 ± 0.43 ^c^

G0, blank control mice; G1, aging mice induced with D-galactose; G2, induced-aging mice treated with metformin; G3, induced-aging mice treated with a low dose (1 g/kg/d) of QBLP; G4, induced-aging mice treated with a high dose (5 g/kg/d) of QBLP. *N* = 6. Different letters indicate significant differences between groups (*p* < 0.05).

## Data Availability

The data presented in this study are available in the article and Supplementary Materials.

## Supplementary Materials

The following supporting information can be downloaded at https://www.mdpi.com/article/10.3390/antiox13111275/s1.

## References

[1] Bana B., Cabreiro F. (2019). The microbiome and aging. Annu. Rev. Genet..

[2] Culig L., Chu X., Bohr V.A. (2022). Neurogenesis in aging and age-related neurodegenerative diseases. Ageing Res. Rev..

[3] Kritsilis M., Rizou S.V., Koutsoudaki P.N., Evangelou K., Gorgoulis V.G., Papadopoulos D. (2018). Ageing, cellular senescence and neurodegenerative disease. Int. J. Mol. Sci..

[4] Nadeeshani H., Li J., Ying T., Zhang B., Lu J. (2022). Nicotinamide mononucleotide (NMN) as an anti-aging health product-promises and safety concerns. J. Adv. Res..

[5] Partridge L., Fuentealba M., Kennedy B.K. (2020). The quest to slow ageing through drug discovery. Nat. Rev. Drug Discov..

[6] Das G., Paramithiotis S., Sivamaruthi B.S., Wijaya C.H., Suharta S., Sanlier N., Shin H.S., Patra J.K. (2020). Traditional fermented foods with anti-aging effect: A concentric review. Food Res. Int..

[7] Zhang Z.Y., Chen X.Y., Loh Y.J., Yang X., Zhang C.H. (2021). The effect of calorie intake, fasting, and dietary composition on metabolic health and gut microbiota in mice. BMC Biol..

[8] Prata G.G.B., Oliveira de Souza K., Lopes M.M.A., Oliveiraet L.S., Aragao F.A.S., Alves R.E., Silva S.M. (2017). Nutritional characterization, bioactive compounds and antioxidant activity of brazilian roses (Rosa spp). J. Agric. Sci. Technol..

[9] Zheng J.Y., Lu B.Y., Xu B.J. (2020). An update on the health benefits promoted by edible flowers and involved mechanisms. Food Chem..

[10] Kim C.S., Cha L.N., Sim M., Jung S., Chun W.Y., Baik H.W., Shin D.M. (2021). Probiotic supplementation improves cognitive function and mood with changes in gut microbiota in community-dwelling older adults: A randomized, double-blind, placebo-controlled, multicenter trial. J. Gerontol. Ser. A.

[11] Zhao T., Wu L.M., Fan F.F., Yang Y.N., Xue X.F. (2022). Supplementation with queen bee larva powder extended the longevity of *Caenorhabditis elegans*. Nutrients.

[12] Haber M., Mishyna M., Martinez J.J.I., Benjamin O. (2019). Edible larvae and pupae of honey bee (*Apis mellifera*): Odor and nutritional characterization as a function of diet. Food Chem..

[13] Tang Q.H., Xiong J., Wang J.X., Cao Z., Liao S.Q., Xiao Y., Tian W.L., Guo J. (2021). Queen bee larva consumption improves sleep disorder and regulates gut microbiota in mice with PCPA-induced insomnia. Food Biosci..

[14] Matsuoka T., Kawashima T., Nakamura T., Kanamaru Y., Yabe T. (2014). Isolation and characterization of proteases that hydrolyze royal jelly proteins from queen bee larvae of the honeybee, Apis mellifera. Apidologie.

[15] Vaiserma A., Romanenko M., Piven L., Moseiko V., Lushchak O., Kryzhanovska N., Guryanov V., Koliada A. (2020). Differences in the gut Firmicutes to Bacteroidetes ratio across age groups in healthy Ukrainian population. BMC Microbiol..

[16] Zhang L., Zhang S.Q., Song H.D., Li B. (2020). Ingestion of collagen hydrolysates alleviates skin chronological aging in an aged mouse model by increasing collagen synthesis. Food Funct..

[17] Miao J.H., Liu J.F., Niu J., Zhang Y.F., Shen W.W., Luo C.W., Liu Y.H., Li C.L., Li H.Y., Yang P.L. (2019). Wnt/β-catenin/RAS signaling mediates age-related renal fibrosis and is associated with mitochondrial dysfunction. Aging Cell..

[18] Liao A.M., Lyu X., Ma J.R., Hou Y.C., Hui M., Liu N., Zhao Y., Cui Y.X., Huang J.H. (2023). Multi-protective effects of wheat embryo globulin on D-gal-induced aging mice. Food Sci. Hum. Wellness.

[19] Anisimov V.N., Berstein L.M., Egormin P.A., Piskunova T.S., Popovich I.G., Zabezhinski M.A., Kovalenko I.G., Poroshina T.E., Semenchenko A.V., Provinciali M. (2005). Effect of metformin on life span and on the development of spontaneous mammary tumors in HER-2/neu transgenic mice. Exp Gerontol..

[20] Kim J.H., Lee S., Cho E.J. (2019). Acer okamotoanum and isoquercitrin improve cognitive function via attenuation of oxidative stress in high fat diet- and amyloid beta-induced mice. Food Funct..

[21] Wang C., Cai Z.X., Wang W., Wei M., Si X.H., Shang Y.T., Yang Z.F., Li T.B., Guo H.S., Li S. (2020). Piperine regulates glycogen synthase kinase-3β-related signaling and attenuates cognitive decline in D-galactose-induced aging mouse model. J. Nutr. Biochem..

[22] Huang H.R., Chen J.J., Chen Y., Xie J.H., Xue P.Y., Ao T.X., Chang X.X., Hu X.B., Yu Q. (2022). Metabonomics combined with 16S rRNA sequencing to elucidate the hypoglycemic effect of dietary fiber from tea residues. Food Sci. Hum. Wellness.

[23] Hou D.Z., Tang J., Huan M.L., Liu F., Zhou S.M., Shen Q. (2022). Alteration of fecal microbiome and metabolome by mung bean coat improves diet-induced non-alcoholic fatty liver disease in mice. Food Sci. Hum. Wellness.

[24] Yin Y.N., Wang J.L. (2021). Predictive functional profiling of microbial communities in fermentative hydrogen production system using PICRUSt. Int. J. Hydrogen Energy.

[25] Machado S., Costa A.S.G., Pimentel B.F., Oliveiran M.B.P.P., Alves R.C. (2020). A study on the protein fraction of coffee silverskin: Protein/non-protein nitrogen and free and total amino acid profiles. Food Chem..

[26] Peixoto J.A.B., Alvarez-Rivera G., Costa A.S.G., Machado S., Cifuentes A., Ibanez E., Oliveiran M.B.P.P., Alves R.C. (2023). Contribution of phenolics and free amino acids on the antioxidant profile of commercial lemon verbena infusions. Antioxidants.

[27] Fan S.J., Yan Y.X., Xia Y., Zhou Z.Y., Luo L.L., Zhu M.N., Han Y.L., Yao D.Q., Zhang L.J., Fang M.L. (2023). Pregnane X receptor agonist nomilin extends lifespan and healthspan in preclinical models through detoxification functions. Nat. Commun..

[28] Wu F., Lei H.H., Chen G., Chen C., Song Y.C., Cao Z., Zhang C., Zhang C., Zhang C., Zhou J.L. (2022). Multiomics analyses reveal that long-term intake of Hesperetin-7-O-glucoside modulates the gut microbiota and bile acid metabolism in mice. J. Agric. Food Chem..

[29] Aruoma O.I., Halliwell B., Hoey B.M., Butler J. (1988). The microbiome and aging. Biochem. J..

[30] Zhang X., Yang Y.P., Su J., Zheng X.J., Wang C.C., Chen S.Q., Liu J.J., Lv L.F., Fan S.H., Zhao A.H. (2021). Age-related compositional changes and correlations of gut microbiome, serum metabolome, and immune factor in rats. Geroscience.

[31] Iakovou E., Kourti M. (2022). A Comprehensive Overview of the Complex Role of Oxidative Stress in Aging, The Contributing Environmental Stressors and Emerging Antioxidant Therapeutic Interventions. Front. Aging Neurosci..

[32] Wu S.J., Lu M.S., Wang S.J. (2017). Antiageing activities of water-soluble chitosan from *Clanis bilineata* larvae. Int. J. Biol. Macromol..

[33] Youn K., Yun E.Y., Lee J., Kim J.Y., Hwang J.S., Jeong W.S., Jun M. (2021). Oleic acid and linoleic acid from *Tenebrio molitor* larvae inhibit BACE1 activity in vitro: Molecular docking studies. J. Med. Food.

[34] Zhang C., Song X.L., Cui W.J., Yang Q.H. (2021). Antioxidant and anti-ageing effects of enzymatic polysaccharide from *Pleurotus eryngii* residue. Int. J. Biol. Macromol..

[35] Doifode T., Giridharan V.V., Generoso J.S., Bhatti G., Collodel A., Schulz P.E., Forlenza O.V., Barichello T. (2021). The impact of the microbiota-gut-brain axis on Alzheimer’s disease pathophysiology. Pharmacol. Res..

[36] Conway J., Duggal N.A. (2021). Ageing of the gut microbiome: Potential influences on immune senescence and inflammageing. Ageing Res. Rev..

[37] Lin S.W., Tsai Y.S., Chen Y.L., Wang M.F., Chen C.C., Lin W.H., Fang T.J. (2021). *Lactobacillus plantarum* GKM3 promotes longevity, memory retention, and reduces brain oxidation stress in SAMP8 mice. Nutrients.

[38] Lopez D.E.G., Lashinger L.M., Weinstock G.M., Bray M.S. (2021). Circadian rhythms and the gut microbiome synchronize the host’s metabolic response to diet. Cell Metab..

[39] Shandilya S., Kumar S., Jha N.K., Kesari K.K., Ruokolainen J. (2022). Interplay of gut microbiota and oxidative stress: Perspective on neurodegeneration and neuroprotection. J. Adv. Res..

[40] Xia C.F., Cao X.Y., Cui L.Y., Liu H., Wang S., Chen T.T. (2020). Anti-aging effect of the combination of *Bifidobacterium longum* and *B. animalis* in a D-galactose-treated mice. J. Funct. Foods.

[41] Cryan J.F., O’Riordan K.J., Cowan C.S.M., Sandhu K.V., Bastiaanssen T.F.S., Boehme M., Codagnone M.G., Cussotto S., Fulling C., Golubeva A.V. (2019). The microbiota-gut-brain axis. Physiol. Rev..

[42] Ding Y., Yan Y.M., Peng Y.J., Chen D., Mi J., Lu L., Luo Q., Li X.Y., Zeng X.X., Cao Y.L. (2019). In vitro digestion under simulated saliva, gastric and small intestinal conditions and fermentation by human gut microbiota of polysaccharides from the fruits of Lycium barbarum. Int. J. Biol. Macromol..

[43] Abreu A.T.A.Y., Milke-Garcia M.P., Arguello-Arevalo G.A., Calderon-de la Barca A.M., Consuelo-Sanchez R.I., Consuelo-Sanchez A., Coss-Adame E., Garcia-Cedillo M.F., Hernandez-Rosiles V., Icaza-Chavez M.E. (2021). Dietary fiber and the microbiota: A narrative review by a group of experts from the Asociación Mexicana de Gastroenterología. Rev. Gastroenterol. Mex..

[44] Zhang C.H., Zhang M.H., Wang S.Y., Han R.J., Cao Y.F., Hua W.Y., Mao Y.J., Zhang X.J., Pang X.Y., Wei C.C. (2010). Interactions between gut microbiota, host genetics and diet relevant to development of metabolic syndromes in mice. ISME J..

[45] Wu X.L., He K., Velickovic T.C., Liu Z.G. (2021). Nutritional, functional, and allergenic properties of silkworm pupae. Food Sci. Nutr..

[46] Xie D.D., Jiang L.Q., Lin Y., Liu Z.W. (2020). Antioxidant activity of selenium-enriched *Chrysomyia megacephala* (Fabricius) larvae powder and its impact on intestinal microflora in D-galactose induced aging mice. BMC Complement. Med. Ther..

[47] Biagi E., Franceschi C., Rampelli S., Severgnini M., Ostan R., Turroni S., Consolandi C., Quercia S., Scurti M., Monti D. (2016). Gut microbiota and extreme longevity. Curr. Biol..

[48] Ponti F., Santoro A., Mercatelli D., Gasperini C., Conte M., Martucci M., Sangiorgi L., Franceschi C., Bazzocchi A. (2020). Aging and imaging assessment of body composition: From fat to facts. Front Endocrinol..

[49] Tavella T., Rampelli S., Guidarelli G., Bazzocchi A., Gasperini C., Pujos-Guillot E., Comte B., Barone M., Biagi E., Candela M. (2021). Elevated gut microbiome abundance of *Christensenellaceae*, *Porphyromonadaceae* and *Rikenellaceae* is associated with reduced visceral adipose tissue and healthier metabolic profile in Italian elderly. Gut Microbes.

[50] Hiippala K., Jouhten H., Ronkainen A., Hartikainen A., Kainulainen V., Jalanka J., Satokari R. (2018). The potential of gut commensals in reinforcing intestinal barrier function and alleviating inflammation. Nutrients.

[51] Yang Q., Liang Q., Balakrishnan B., Belobrajdic D.P., Feng Q.J., Zhang W. (2020). Role of dietary nutrients in the modulation of gut microbiota: A narrative review. Nutrients.

[52] Machiels K., Joossens M., Sabino J., De Preter V., Arijs I., Eeckhaut V., Ballet V., Claes K., Van Immerseel F., Verbeke K. (2014). A decrease of the butyrate-producing species *Roseburia hominis* and *Faecalibacterium prausnitzii* defines dysbiosis in patients with ulcerative colitis. Gut.

[53] Liu M.Y., Yun S.J., Cao J.L., Cheng F.E., Chang M.C., Meng J.L., Liu J.Y., Cheng Y.F., Xu L.J., Geng X.R. (2021). The fermentation characteristics of Sparassis crispa polysaccharides and their effects on the intestinal microbes in mice. Chem. Biol. Technol. Agric..

[54] Li P., Lu B.Y., Gong J., Li L., Chen G.P., Zhang J.X., Chen Y.D., Tian X., Han B., Guo Y.K. (2021). Chickpea extract ameliorates metabolic syndrome symptoms via restoring intestinal ecology and metabolic profile in type 2 diabetic rats. Mol. Nutr. Food Res..

[55] Fujita S., Volpi E. (2006). Amino acids and muscle loss with aging. J. Nutr..

[56] Xi W., Song N., Yan Q., Liang H., Zhang W. (2021). The analysis of the effects of Liuwei Dihuang decoction on aging-related metabolites and metabolic pathways in naturally aging mice by ultra-performance liquid chromatography quadruple time-of-light mass spectrometry. J. Physiol. Pharmacol..

[57] Zhao S.J., Liu X.J., Tian J.S., Gao X.X., Liu H.L., Du G.H., Qin X.M. (2020). Effects of Guilingji on aging rats and its underlying mechanisms. Rejuvenation Res..

[58] Krishnamurthy K., Laskowitz D.T. (2015). Cellular and molecular mechanisms of secondary neuronal injury following traumatic brain injury. Transl. Res. Trauma. Brain Inj..

[59] Ren M., Zhang S.H., Zeng X.F., Liu H., Qiao S.Y. (2015). Branched-chain amino acids are beneficial to maintain growth performance and intestinal immune-related function in weaned piglets fed protein restricted diet. Asian-Australas J. Anim. Sci..

[60] Parkhitko A.A., Ramesh D., Wang L., Leshchiner D., Filine E., Binari R., Olsen A.L., Asara J.M., Cracan V., Rabinowitz J.D. (2020). Downregulation of the tyrosine degradation pathway extends *Drosophila* lifespan. Elife.

[61] Nguyen L., Rigo J.M., Rocher V., Belachew S., Malgrange B., Rogister B., Leprince P., Moonen G. (2001). Neurotransmitters as early signals for central nervous system development. Cell Tissue Res..

