# Supplementary material for: Queen Bee Larva, an Edible By-Product of Royal Jelly, Alleviate D-Galactose-Induced Aging in Mouse by Regulating Gut Microbiota Structure and Amino Acid Metabolism
Source: Antioxidants (Basel). 2024 Oct 22;13(11):1275. doi: 10.3390/antiox13111275 (PMC11591118; doi:10.3390/antiox13111275)
Supplement: Supplementary file 1 [file antioxidants-13-01275-s001.zip › antioxidants-3234272-supplementary.pdf]

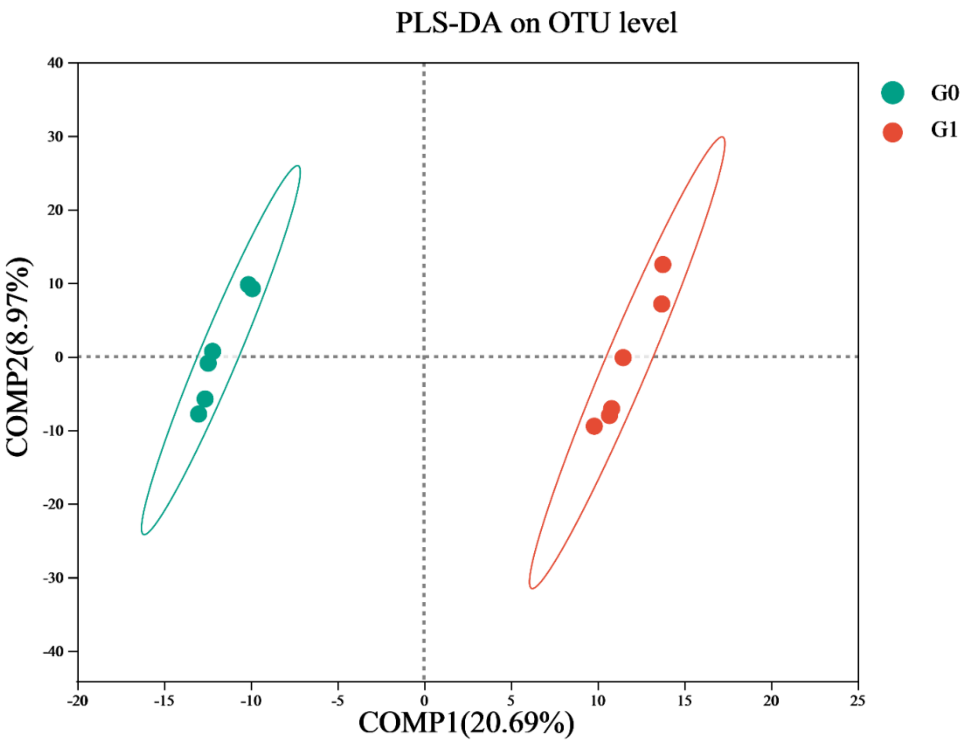

Figure S1. Partial least squares discriminant analysis (PLS-DA) score plot generated by using the abundance profile of OTUs of G0 and G1. G0, blank control; G1, aging mouse model induced with D-galactose.

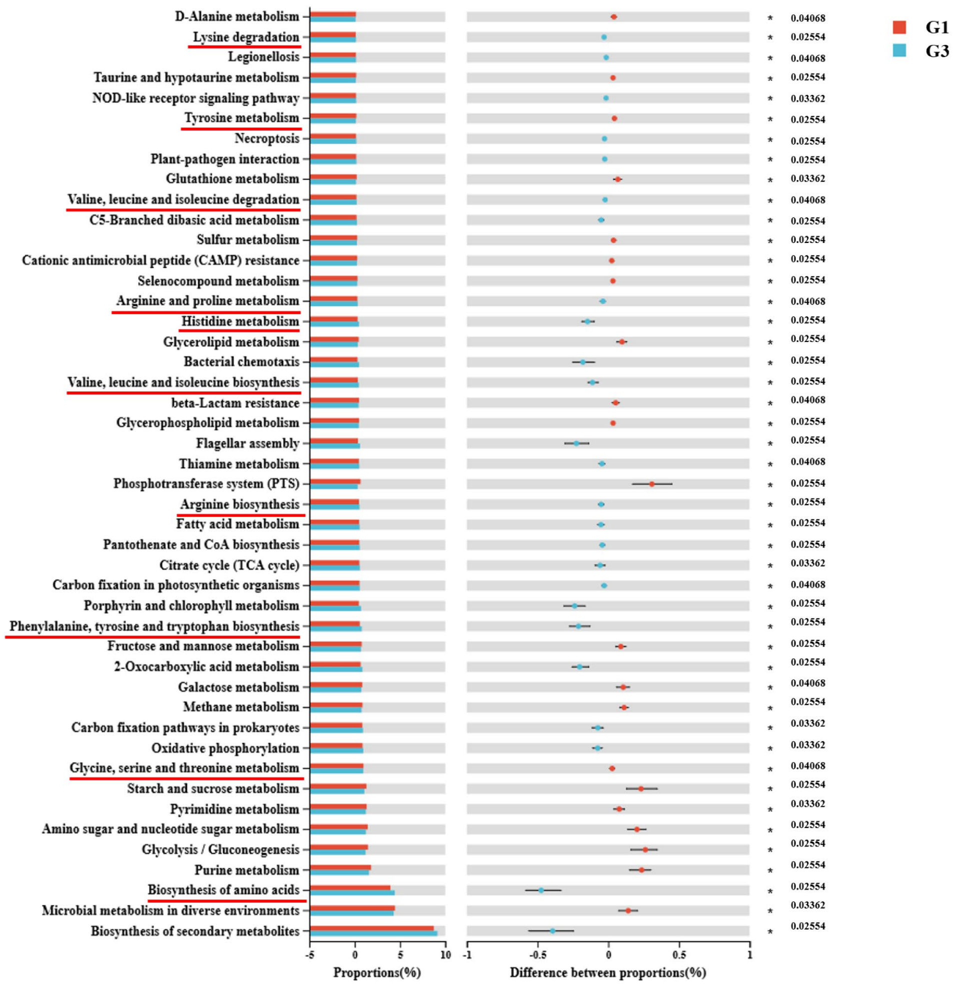

Figure S2. Predicted metabolic pathways in the gut microbiota in a mouse model of aging. G1, aging mouse model induced with D-galactose. G3, induced aging mouse model treated with queen bee larva powder (QBLP). Underline indicates that pathways involve amino acids.

Table S1. Cross-validation with CV-ANOVA values of OPLS-DA models obtained from UPLC-Q-Exactive data of samples of G0 and G1, G3 and G1. G0, blank control; G1, aging mouse model induced with D-galactose; G3, induced aging mouse model treated with queen bee larva powder (QBLP).

**Groups**	***p* Values**
G0 vs. G1	0.04
G3 vs. G1	0.02
